# CD300A promotes tumor progression by PECAM1, ADCY7 and AKT pathway in acute myeloid leukemia

**DOI:** 10.18632/oncotarget.24164

**Published:** 2018-01-11

**Authors:** Xiaogang Sun, Shuhong Huang, Xin Wang, Xiaohua Zhang, Xin Wang

**Affiliations:** ^1^ Shandong Provincial Hospital Affiliated to Shandong University, Jinan, P.R. China; ^2^ Department of Neurobiology, Key Laboratory of Medical Neurobiology, School of Medicine, Shandong University, Jinan, Shandong, P.R. China; ^3^ Tengzhou Municipal Hospital of Traditional Chinese Medicine, Tengzhou, Shandong, P.R. China

**Keywords:** CD300A, PECAM1, ADCY7, acute myeloid leukemia, U937

## Abstract

CD300A is a member of the CD300 glycoprotein family of cell surface proteins involved in immune response signaling pathways. There is evidence that CD300A plays a role in autophagy and angiogenesis, while, no studies have been reported which investigated the role of CD300A in tumors. CD300A was found to be highly expressed with statistical significance in acute myeloid leukemia (AML), as well as associated with prognosis, through the analysis of differential expression genes using the TCGA and GTEx database. A decrease in CD300A expression could promote apoptosis and inhibit proliferation and migration of AML cell line U937, as well as promote the activation of the AKT/mTOR pathway. These results demonstrated that CD300A operated as a tumor promoter in AML cells. We further analyzed coexpression genes of CD300A and then screened two genes, ADCY7 and PECAM1, which were both overexpressed and associated with poor prognosis in AML. Meanwhile, CD300A increased the expression of PECAM1 and ADCY7 in U937 cells. Furthermore, we demonstrated that PECAM1 promoted the proliferation and migration and inhibited the apoptosis of U937 cells. ADCY7 participated in the regulation of proliferation and migration, but not apoptosis, in U937 cells. Both PECAM1 and ADCY7 promoted tumor progression through the AKT pathway, showing the same molecular mechanism as CD300A. To summarize, we, for the first time, confirmed that CD300A promoted tumor progression by increase PECAM1 and ADCY7 expression, and activating the AKT/mTOR signaling pathway in AML. It is suggested CD300A is an oncogene and potential therapeutic target for AML.

## INTRODUCTION

Acute myeloid leukemia (AML) belongs to a group of hematological malignancies originating from hematopoietic stem cells [1, 2]. In recent years, many proto-oncogenes and tumor suppressor genes have been discovered in blood tumors, and genomics researches have greatly promoted the discovery and clinical application of molecular markers. With the application of whole genome sequencing in human malignant tumors, the frequency of discovering differentially expressed genes continues to improve [3, 4]. Although cytogenetics is an independent prognostic marker for AML, more than half of AML lacks characteristic chromosomal changes [5]. Studies of tumor related marker genes are necessary for understanding the molecular mechanism of AML progression and provide the basis for further individualized therapy and targeted therapy.

While studying prophase, CD300A was found to be highly expressed with statistical significance in AML, as well as associated with prognosis, through analysis of differential expression genes using the TCGA and GTEx database. CD300A encodes a member of the CD300 glycoprotein family of cell surface proteins found on leukocytes involved in immune response signaling pathways [6]. This gene is located on chromosome 17 in a cluster with all but one of the other family members. Multiple transcript variants encoding different isoforms have been found for this gene. CD300A plays a role in autophagy and angiogenesis, but prior studies that have noted the importance of CD300A in tumors [7–9].

The present study was designed to determine the function and molecular mechanism of CD300A in AML, as well as two CD300A coexpression genes, ADCY7 and PECAM1. We demonstrated that CD300A regulates the proliferation and apoptosis of AML cells as a tumor promoter, playing a positive role in AML as a potential therapeutic target.

## RESULTS

### CD300A is over-expression and predicts prognosis in AML

**Figure 1:**
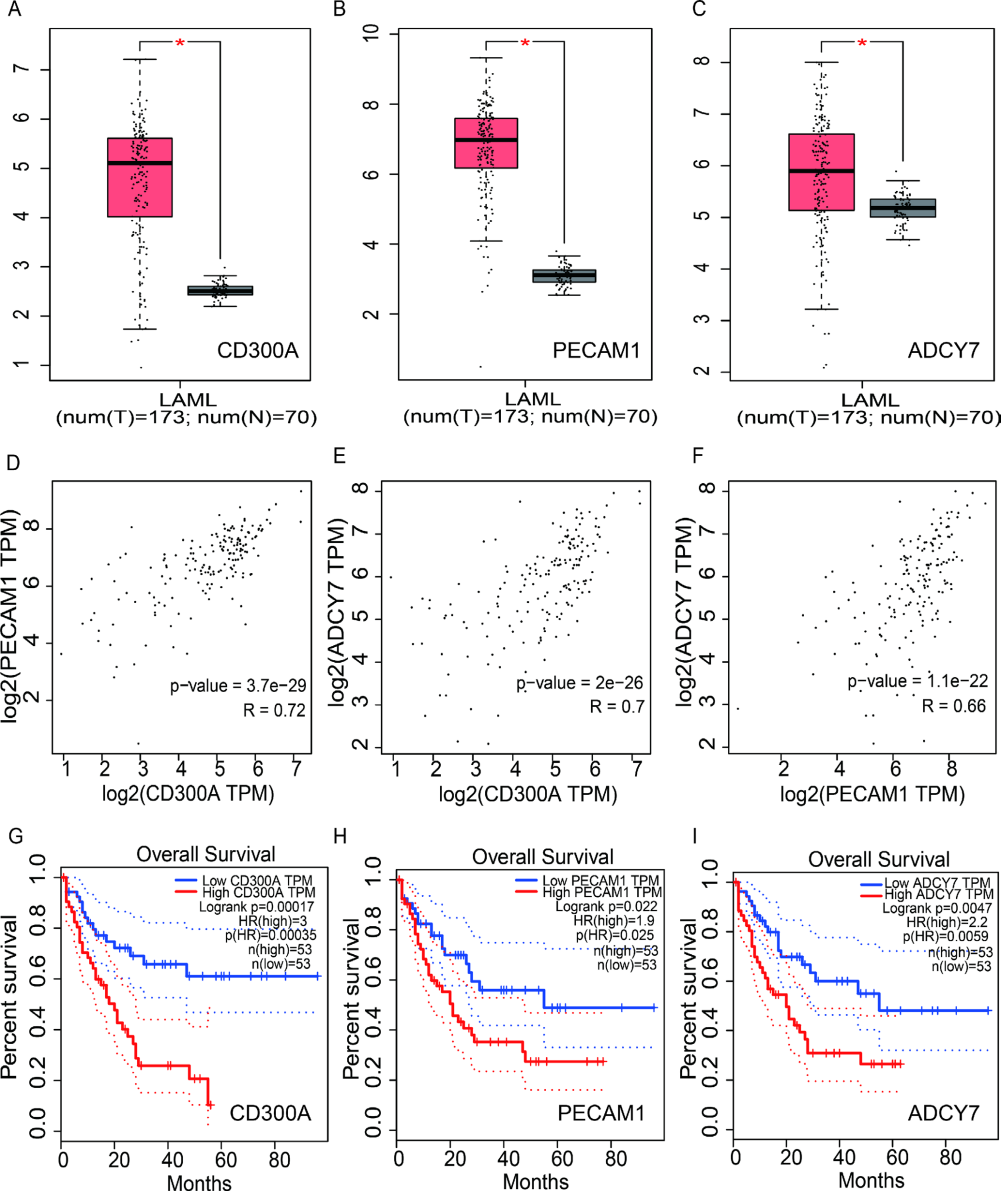
Analysis of the expression and prognosis value of CD300A, PECAM1 and ADCY7 in human acute myeloid leukemia (**A**–**C**) The mRNA expression of CD300A, PECAM1, and ADCY7, were all significantly higher compared to the normal sample. (**D**–**F**) Representing CD300A, PECAM1, and ADCY7, showed coexpression in AML. In graphs (**G**–**I**) low mRNA expression of CD300A was correlated with a better prognosis in AML, as well as PECAM1 and ADCY7. In conclusion, these results suggest that CD300A, PECAM1, and ADCY7 have functional relevance and potential biological functions in AML.

In recent years, with the development of High-throughput RNA sequencing (RNA-Seq), large amounts of RNA-Seq data have emerged. Gene Expression Profiling Interactive Analysis (GEPIA) is an online tool based on the Cancer Genome Atlas (TCGA) and Genotype-Tissue Expression (GTEx) dataset for transcriptomic analysis [10]. To evaluate the expression and prognosis role of CD300A in AML, we analyzed this gene on GEPIA. As shown in Figure 1A, CD300A mRNA level was significantly higher in AML (*n* = 173) compared to the normal sample (*n* = 70). The survival curves demonstrated a significant correlation between patients with high CD300A expression levels and a poor prognosis. Moreover, two genes, ADCY7 and PECAM1, with high correlations to CD300A expression levels were screened out, which also had potential prognostic values in AML by coexpression analysis. Both ADCY7 and PECAM1 were up-regulated in AML, and patients with high ADCY7 and PECAM1 expression levels were both associated with poorer overall survival (Figure 1B–1CB, 1H–1IH). Furthermore, there was a significant correlation between CD300A and PECAM1 (R = 0.72, *P* < 0.05), as well as ADCY7 (R = 0.7, *P* < 0.05) (Figure 1D–1F). In general, results of dataset analysis suggest that the three genes may interact with each other and promote the development of AML, providing us with directions for further research.

### CD300A increased ADCY7 and PECAM1 expression in U937 cells

**Figure 2:**
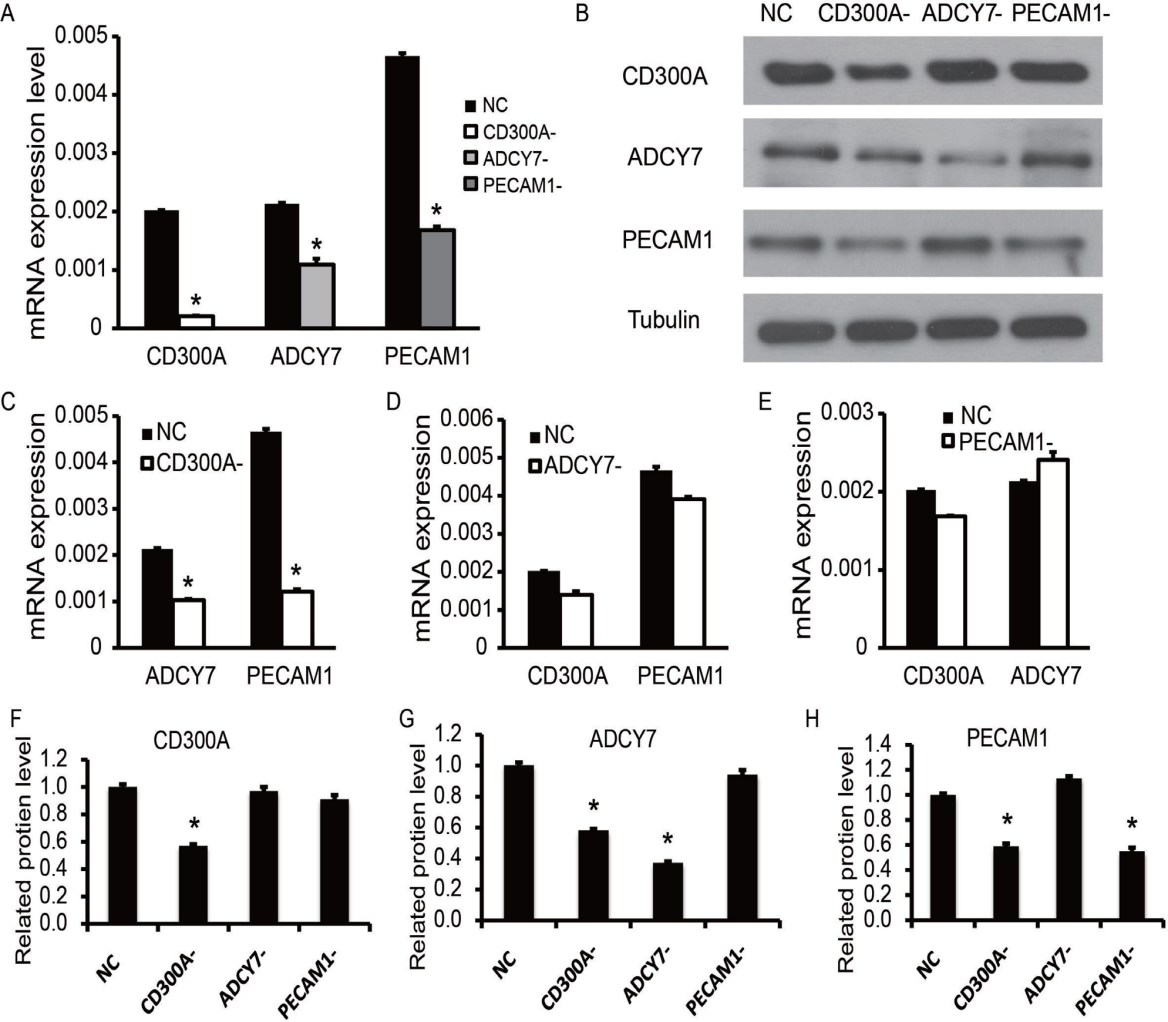
Coexpression detection of CD300A, PECAM1, and ADCY7 in AML cell line U937 (**A**) The knockdown efficiencies of CD300A, PECAM1, and ADCY7 were detected by RT-PCR, which indicated the expression of these three genes was significantly decreased in each knockdown group. (**C**) ADCY7 and PECAM1 were significantly down-regulated in the CD300A knockdown group. (**D**) The expression of CD300A showed no significant difference between NC and ADCY7- groups, as well as PECAM1. (**E**) The expression of CD300A showed no significant difference between NC and PECAM1- groups, as well as ADCY7. (**B**, **F**–**H**) Western blot analysis was performed to detect the expression of these three genes in each group based on protein level. F, CD300A only showed a significant decrease in group CD300A-. G, ADCY7 showed a significant decrease in groups ADCY7- and CD300A-. H, PECAM1 showed significant decreases in groups PECAM1- and CD300A-. These results suggest that the knockdown of CD300A caused a decrease in expression levels of ADCY7 and PECAM1.

To find out whether the coexpression of CD300A, ADCY7, and PECAM1 in AML is due to mutual accommodation, we first designed the shRNA of CD300A, ADCY7 and PECAM1 with significant inhibitory efficiency (Figure 2A). Total RNA and proteins of each group were collected for testing gene expression. Results of Western blotting (Figure 2G–2HG) and qPCR (Figure 2C–2E) showed that the expression of ADCY7 and PECAM1 was significantly decreased during CD300A inhibition. In contrast, the expression of CD300A did not change significantly in the ADCY7 and PECAM1 inhibitory groups. According to these data, we can infer that CD300A positively regulates the expression of ADCY7 and PECAM1 in U937 cells.

### CD300A promotes the proliferation and migration of U937 cells

**Figure 3:**
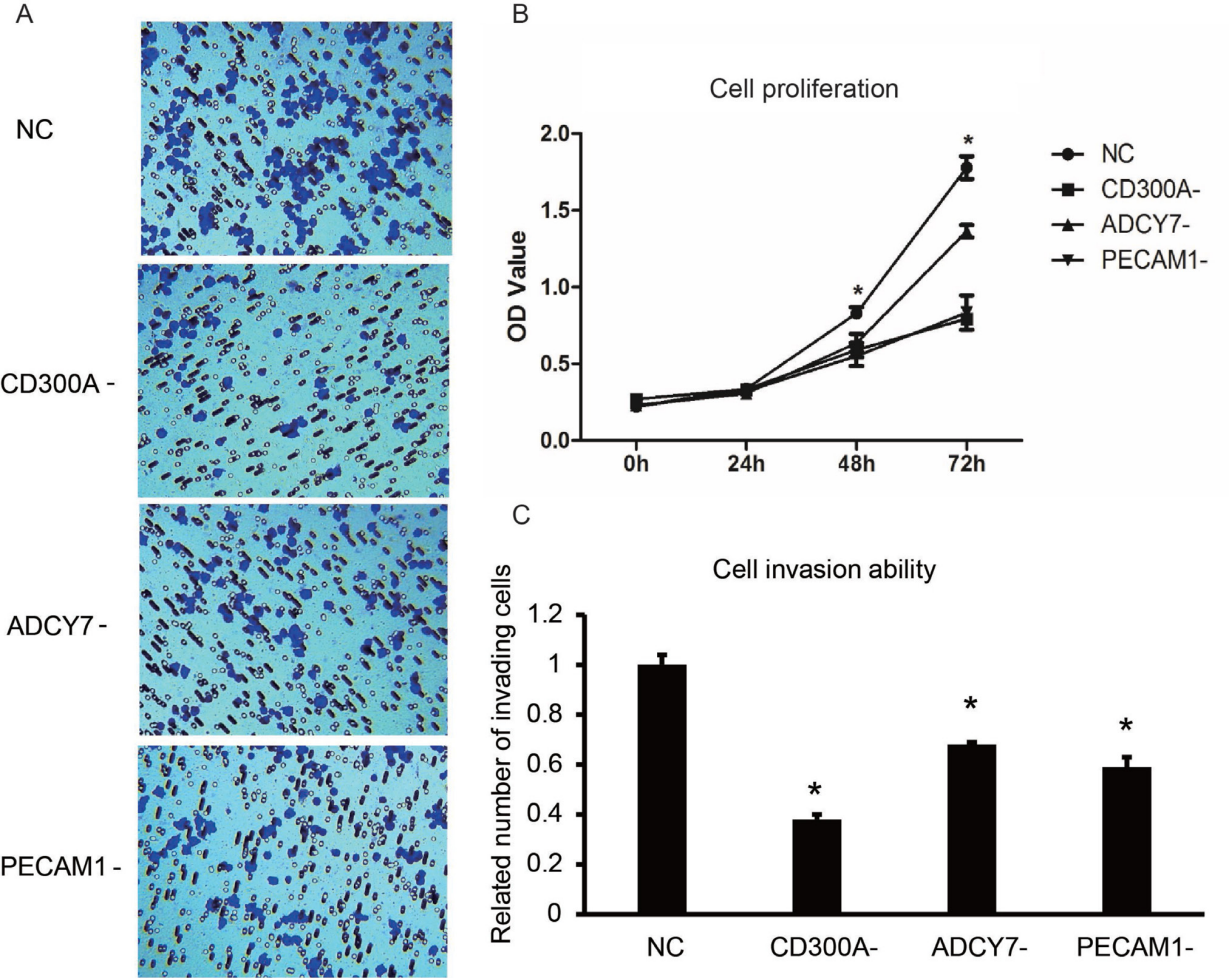
Effects of CD300A, PECAM1, and ADCY7 on proliferation and invasion in U937 cells (**A**–**C**) Transwell assay was performed to detect the effects of CD300A, PECAM1, and ADCY7 on invasion in U937 cells. Compared to the NC group, related numbers of invading cells of CD300A-, ADCY7- and PECAM1- were significantly decreased. (B) CCK8 was performed to detect the proliferation of each group in U937 cells. Compared to the NC group, the proliferation rates of CD300A,- ADCY7- and PECAM1 decreased significantly.

The dysfunction of cell proliferation, invasion and apoptosis are the crucial mutations that promote tumor development. Therefore, the analysis of the target gene functions on biological behavior of tumor cells is crucial to the research of tumor gene function. At present, CD300A function in AML remains unknown, so we need to first elucidate the biological behaviors of AML cells that CD300A is involved in. The effects of CD300A, ADCY7 and PECAM1 on the proliferation of U937 cells were investigated by CCK8. As shown in Figure 3, the cell proliferation rate of CD300A-, ADCY7- and PECAM1- groups decreased significantly compared to the NC group, while the decrease in CD300A- and PECAM1- groups was more significant than that in ADCY7-, suggesting a more effective role of CD300A and PECAM1 on cell proliferation (Figure 3B). We further examined the migration ability of the cells in each group by transwell. Compared with the NC group, the migration ability of CD300A-, ADCY7- and PECAM1- group cells decreased significantly, while the decrease in CD300A- and PECAM1- groups was more obvious (Figure 3A–3C). These results suggest that CD300A, PECAM1 and ADCY7 promote the migration of U937 cells.

### CD300A inhibits apoptosis of U937 cells by PECAM1

**Figure 4:**
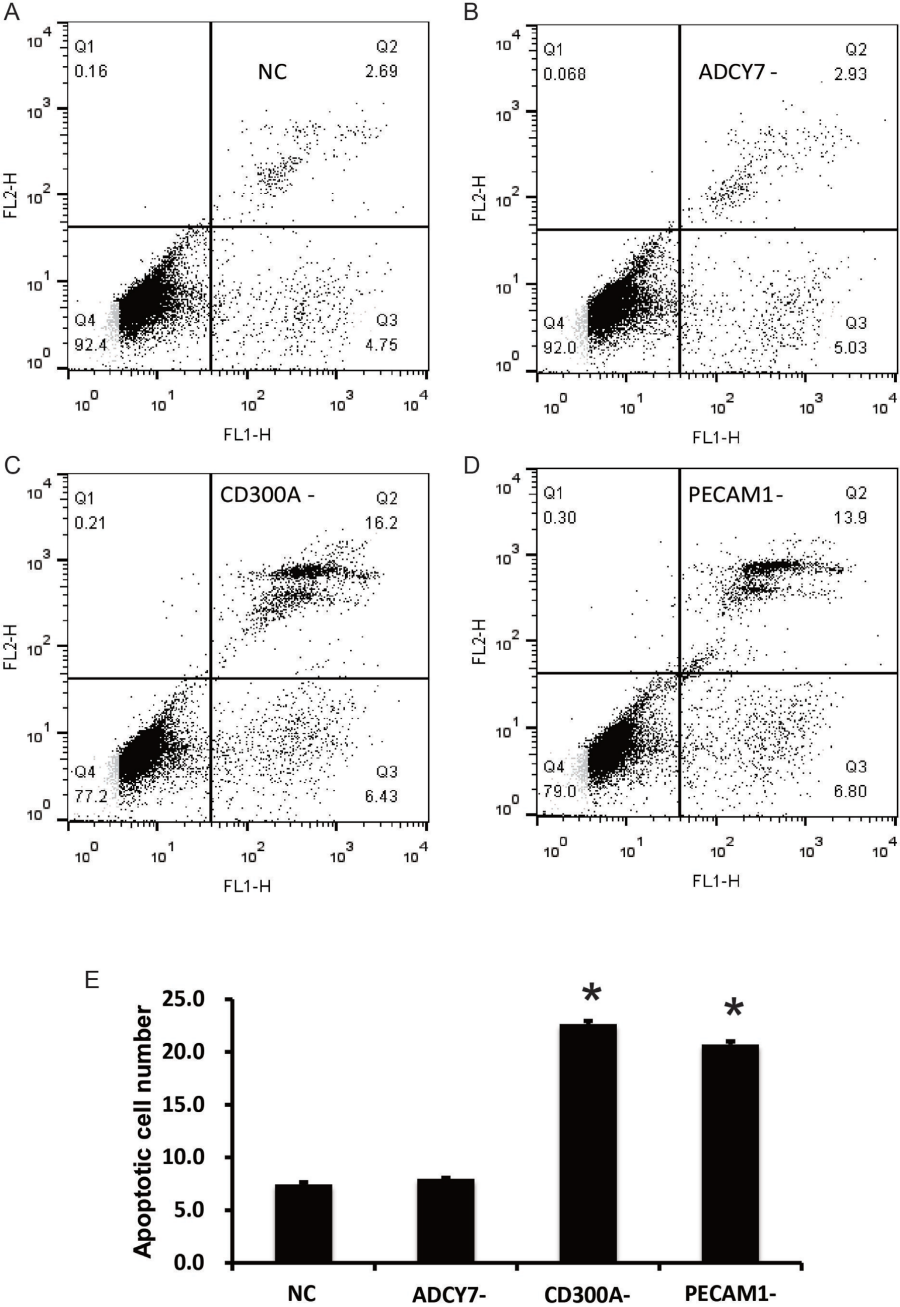
Effects of CD300A, PECAM1, and ADCY7 on apoptosis in U937 cells (**A**–**D**) Flow Cytometry Analysis was conducted for apoptosis determination. (**E**) Apoptosis cell numbers of CD300A- (22.63%) and PECAM1- (20.7%) groups were significantly increased compared to the NC group (7.44%), but there were no significant changes in the ADCY7- group (7.96%).

**Figure 5:**
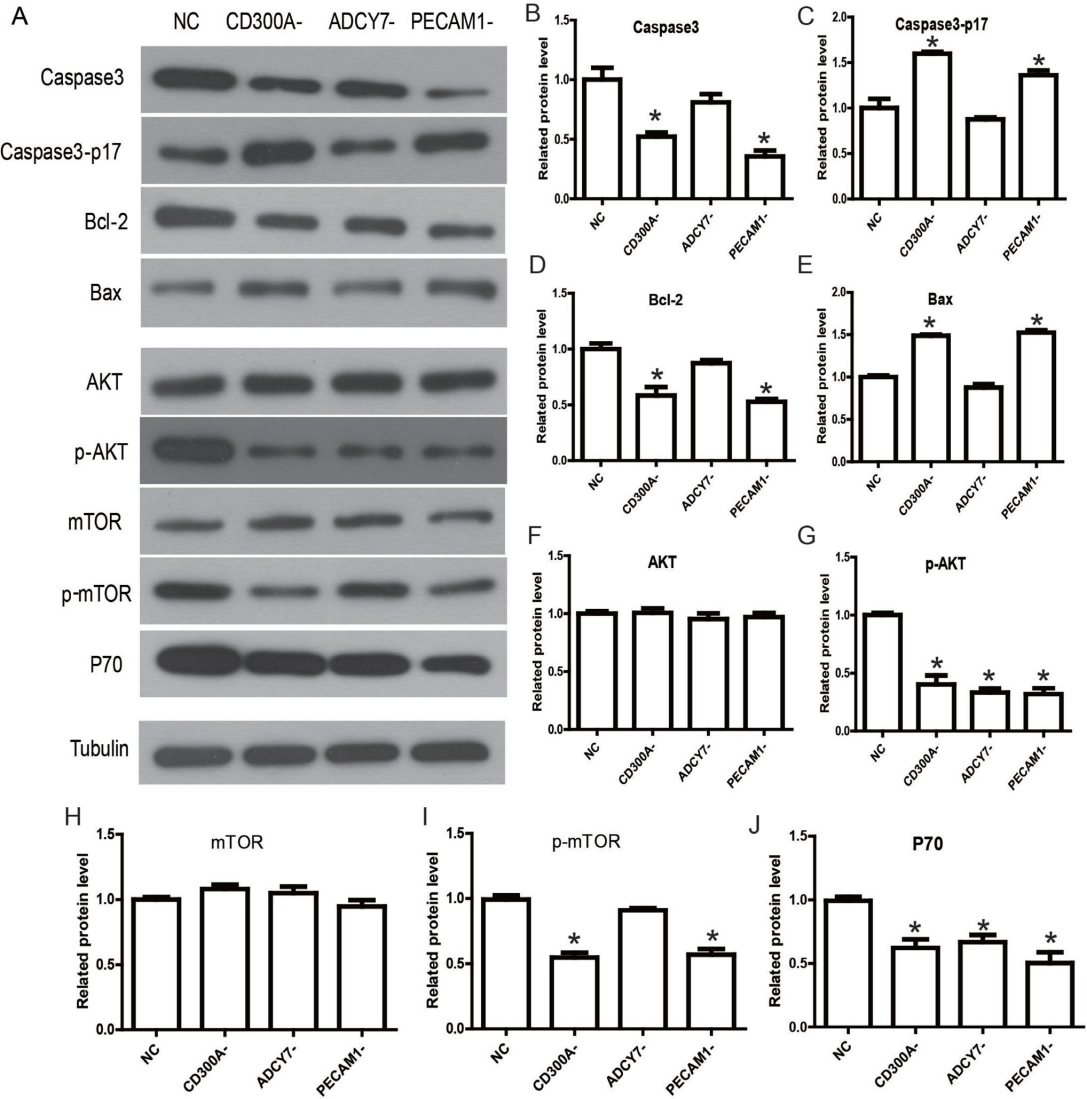
Determination of the molecular mechanisms and signaling pathways by which CD300A regulates apoptosis and proliferation in U937 (**A**) Western blot analysis was performed to detect the related gene expression of signaling pathway. (**B**–**E**) Apoptosis factor, caspase3, was specifically activated in CD300A- and PECAM1- groups. Meanwhile, Bcl-2 expression was inhibited in CD300A- and PECAM1- groups, while Bax was activated. (**F**–**J**) Phosphorylation levels of AKT and mTOR decreased under unchanged levels of total AKT and mTOR in CD300A- and PECAM1- groups. The expression of P70 was down-regulated in CD300A-, ADCY7- and PECAM1- groups.

Flow cytometry was performed to detect the apoptosis of U937 cells. The results showed that, compared with the NC group (7.44%), the number of apoptotic cells in the CD300A- (22.63%) and PECAM1- groups (20.7%) increased significantly, but no significant differences were found in the ADCY7- group (7.96%) (Figure 4). These results suggest that CD300A and PECAM1 can inhibit the apoptosis of U937 cells, and CD300A may play an inhibitory role in apoptosis by PECAM1. According to our previous results, which suggest that CD300A can regulate the expression of PECAM1, we hypothesized that CD300A may inhibit apoptosis by increasing PECAM1 expression. Further, Western blot analysis was performed to detect the expression of apoptosis related proteins in each group to confirm the mechanism by which CD300A and PECAM1 inhibit cell apoptosis. As shown in Figure 5, the apoptosis factor, Caspase3, was specifically activated in CD300A- and PECAM1- groups. Meanwhile, the apoptosis inhibiting gene, Bcl-2, was decreased in CD300A- and PECAM1- groups, while the apoptosis promoting gene, Bax, was activated. These findings suggest that CD300A and PECAM1 inhibit the apoptosis by regulating the activity of Bcl-2 and Bax in U937 cells.

### CD300A regulated apoptosis and proliferation of U937 cells through PECAM1 and AKT pathways

**Figure 6:**
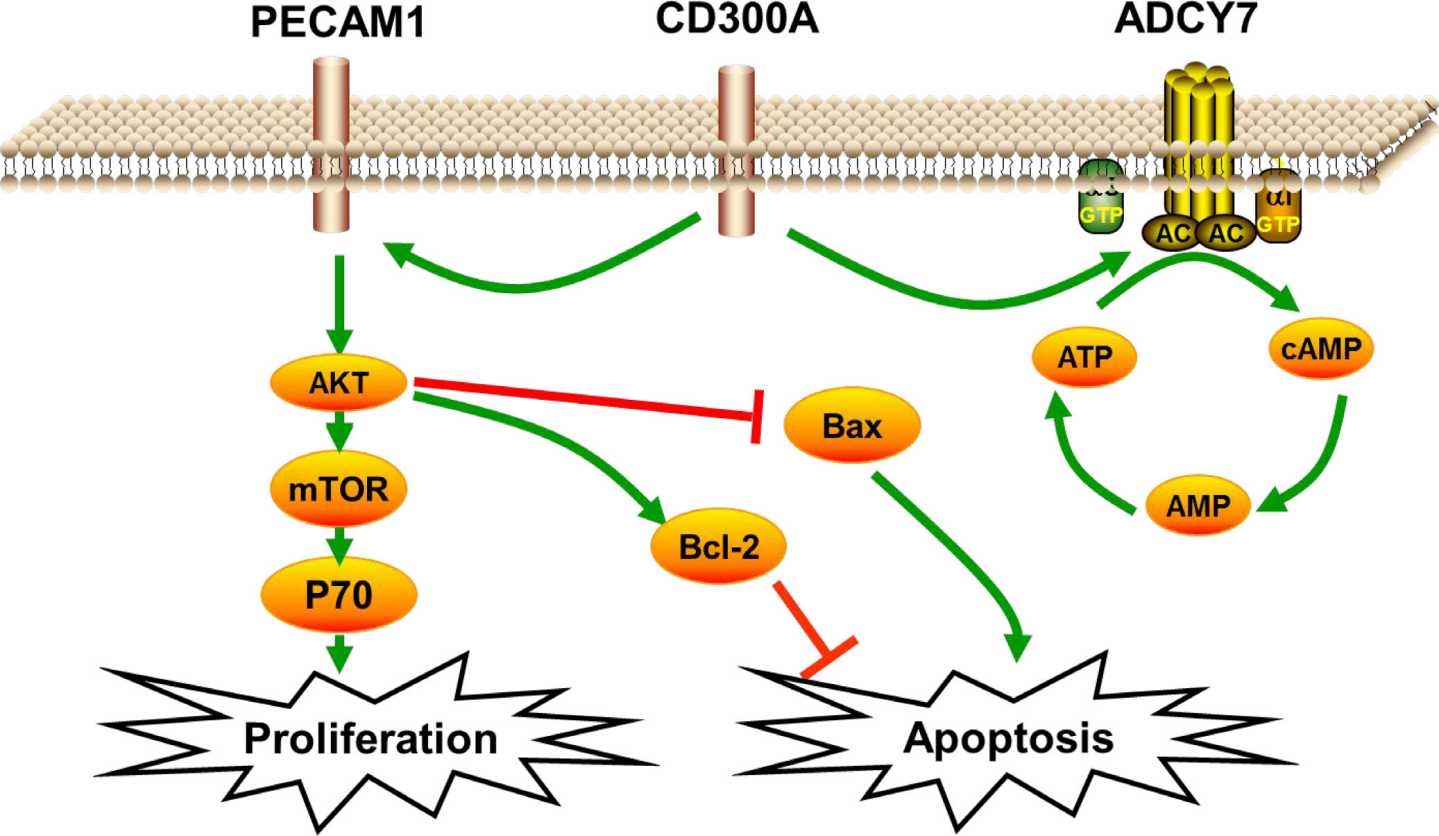
Schematic diagram of CD300A/PECAM1/AKT signaling pathway on the proliferation and apoptosis of AML cells CD300A regulated PECAM1 expression, then activated the AKT/mTOR pathway, up-regulated P70 to promote proliferation, and at the same time down-regulated the apoptosis factor to inhibit apoptosis in human AML.

We detected the possible relevant signaling pathways to elucidate the specific mechanisms by which CD300A promotes AML progression through Western blot analysis. The AKT pathway is a frequently activated pathway in genesis and development of tumors [11]. It regulates the proliferation, apoptosis and migration of tumor cells, and has also been reported in AML [12]. Therefore, we predicted that CD300A modulates the AKT pathway and thereby affects tumor progression. So, the key genes of the AKT pathway were selected to elucidate the mechanism of CD300A in AML. As shown in Figure 5, phosphorylation levels of AKT and mTOR decreased under unchanged levels of total AKT and mTOR in CD300A- and PECAM1- groups. The expression of P70 was down-regulated in CD300A-, ADCY7- and PECAM1- groups. According to the above results, the CD300A/PECAM1/AKT signaling pathway on the proliferation and apoptosis of AML cells was plotted in Figure 6. CD300A regulated PECAM1 expression, and then activated the AKT/mTOR pathway, and up-regulated P70 to promote proliferation. At the same time, CD300A down-regulated the apoptosis factor to inhibit apoptosis in human AML.

## DISCUSSION

In the present study, CD300A was found to be up-regulated and associated with prognosis in AML by gene expression analysis of an online database (Figure 1A, 1G). CD300A is an inhibitory receptor of the CD300 glycoprotein family, with a molecular weight of about 60KD [13]. Similar to the family structure, CD300A is a transmembrane protein, with a cytoplasmic caudal region including four ITIMs domains. The binding of CD300A to ligands leads to phosphorylation of tyrosine residues in ITIMs domains [14]. After phosphorylation, ITIMs is able to recruit different phosphatases to transmit the signals in the cell, including SHIP, SHP-1, and SHP-2, which are all members of protein tyrosine phosphatases (PTP) [15]. In 2015, L Jiang et al. demonstrated that the suppression of CD300A inhibited the growth of diffuse large B-cell lymphoma and also lead to a reduction of p-AKT levels [16]. In another research, patient-derived acute lymphoblastic leukemia (ALL) cells expressed CD300A at high levels compared to normal pre-B cells and the patients with high CD300A levels showed worse OS compared to the others [17]. The results of this study showed that CD300A knockdown inhibited proliferation and migration and promoted apoptosis in AML cell line U937, suggesting CD300A functions as a tumor promoter in AML (Figure 3A, 3B and Figure 4D). Moreover, inhibition of CD300A resulted in a decrease in phosphorylation of AKT and mTOR (Figure 5). According to these foundings, we predicted that CD300A affected the activation of AKT/mTOR signaling pathways by recruiting PTP, thereby regulating the expression of proliferation and apoptosis related genes and affecting the biological behavior of tumor cells.

However, does CD300A have other mechanisms or target proteins in the regulation of tumor cell function? We analyzed the coexpression gene of CD300A in AML by online database and screened two CD300A coexpression genes, ADCY7 and PECAM1, which were both overexpressed and associated with prognosis in AML (Figure 1B–1FB, 1H–1IH). In 1995, K Hellevuo et al. discovered a novel form of human adenylyl cyclase (ADCY7) in the human erythroleukemia cell line (HEL) and localized this gene on 16q12–16q13, which was characterized by 12 membrane-spanning domains [18]. C Li et al have recently demonstrated that ADCY7 inhibition resulted in elevated apoptosis, decreased cell growth and lower c-Myc expression, suggesting that G protein-coupled receptor signaling regulated the genesis and development of AML [19]. Platelet endothelial cell adhesion molecule 1 (PECAM1), also known as CD31, is a member of the adhesion molecule in the immunoglobulin superfamily. The coding gene of PECAM1 located at the end of the long arm of chromosome 17 [20]. Several reports show that PECAM1 is a type I transmembrane glycoprotein, expressed mainly in monocytes, platelets, endothelial cells, discrete circulating lymphocytes, and polymorph nuclear cells. Because all vascular endothelial cells in developing and mature individuals contain high levels of PECAM1, it is used as a marker of vascular endothelial cells [21]. PECAM1 can mediate cell adhesion by activation of integrin, platelet regulation, leukocyte endothelial cell migration, apoptosis inhibition and signal transduction. Previous studies have shown that PECAM1 is associated with metastasis and progression of solid tumors, including gastric cancer, lung cancer, and glioma, melanoma and breast cancer [22–26]. In hematological malignancies, PECAM1 also acts as a cancer promoter. Elevated levels of sPECAM-1 were detected in serum of patients with chronic myelogenic leukemia. A recent study demonstrated that PECAM1 may down-regulate imatinib-induced apoptosis of Philadelphia chromosome-positive leukemia cells by BCR/ABL signaling [27]. In AML cells, PECAM-1 and CD38 coexpression levels regulated the extramedullary dissemination [28].

In this study, we investigated the role of PECAM1 and ADCY7 in AML. PECAM1 promoted proliferation and migration and inhibited the apoptosis, and ADCY7 participated in the regulation of proliferation and migration but not apoptosis of U937 cells (Figures 3A, 3B). Both PECAM1 and ADCY7 promote tumor progression through the AKT pathway, showing the same molecular mechanism as CD300A (Figure 5). Furthermore, CD300A knockdown inhibited PECAM1 and ADCY7 expression in U937 cells, implying a potential pathway of CD300A in AML cells. As cell surface proteins, CD300A has been proved to regulate AKT downstream signaling pathway. According to those results, we predicted that CD300A targeted PECAM1 and ADCY7, which activated the AKT pathway and then promoted the proliferation in U937 cells. However, it is interesting to note that CD300A inhibits the apoptosis through PECAM1 but not ADCY7 in U937 cells, implying the unknown mechanisms by which CD300A promotes tumor progression. In briefly, CD300A may contribute to tumor proliferation and apoptosis by upregulating PECAM1 and ADCY7, in addition to recruiting PTP in AML cells (Figure 6). In conclusion-we, for the first time, confirme that CD300A promotes cell proliferation and inhibits apoptosis of human AML by increasing PECAM1 and ADCY7 expression and AKT/mTOR signaling pathway activity. The results of this investigation show that CD300A was a potential therapeutic target for AML and requires further clinical trials to validate.

## MATERIALS AND METHODS

### Cell lines and cell cultures

Human acute myeloid leukemia cell line U937 was presented by the Basic Medical College of Shandong University, Jinan, China. U937 cells were maintained in RPMI 1640 medium containing 10% fetal bovine serum, 100 units/mL penicillin and 100 units/mL streptomycin (Invitrogen Corp.) at 37°C in a humidified atmosphere with 5% CO_2_.

### Real-time quantitative PCR (qPCR)

The total RNA of each sample was reverse transcribed using the Reverse Transcription Reaction Kit (from CWBIO) according to the manufacturer′s instructions. qPCR was performed on an FTC-3000 Real-Time PCR System to determine the relative amounts of CD300A, ADCY7, PECAM1 and β-actin (internal control) mRNA expressed. Primers used for qPCR were as follows: CD300A sense: 5′- TGGGCTCTGTTGCTTCTCTG-3′ and antisense: 5′- GCCGTTCCTTTTTCCTGCTG -3′; PECAM1 sense: 5′- GTCAAGCCTCAGCACCAGAT-3′ and antisense: 5′-CACCTGGTACTCTGCAGTGG -3′; ADCY7 sense: 5′- AGAGGCACCAGAATGTCAGC-3′ and antisense: 5′-CAGTAGTAGCAGTCGCCGAG-3′. The relative amount of mRNA was calculated using the comparative CT method after normalization to the β-actin mRNA levels.

### Western blotting and antibody

The total protein content (44 μg) from each sample was resolved by 10% SDS-PAGE, and then transferred to a PVDF membrane. The membrane was blocked by 5% fat-free milk in TBS containing 0.1% Tween-20 for 1 hour at room temperature. Next, it was incubated with primary antibodies at a dilution of 1:1000 for 1 hour and with the secondary antibodies at a dilution of 1:5000 for 1 hour. Primary antibodies used for Western blotting were as follows: CD300A (abcam), PECAM1 (proteintech), ADCY7 (GeneTex), tubulin (proteintech), caspase3 (proteintech), Bcl-2 (proteintech), Bax (proteintech), AKT (proteintech), p-AKT (proteintech), mTOR (Cell Signaling Technology), p-mTOR (Cell Signaling Technology), and p70 (proteintech). All assays were repeated three times. The quantification analyses were performed using Image J. The relative expression levels of proteins were calculated by determining a ratio between the amount of protein and tubulin.

### Cell proliferation assay

Cell proliferation assay was performed to detect the proliferation by Cell Counting Kit-8 (CCK-8). The U937 cells were divided into four groups: negative control (NC), CD300A knockdown (CD300A-), ADCY7 knockdown (ADCY7-) and PECAM1 knockdown (PECAM1-). The U937 cells were plated in a 96-well plate at a concentration of 1000 cells per well. The cell proliferation assay was performed every 24 hours as follows: 10 μL of CCK-8 solution (from Solarbio) was added to each well, and the plate was then incubated at 37°C for 2 hours. The absorbance was measured at a wavelength of 450 nm using a microplate reader.

### Transwell assay

Transwell assay was performed using Transwell chambers (Merck Millipore) according to the manufacturer′s instructions. The cells were grouped the same as the CCK-8 assay. 100 μL cells suspension (about 5×10^4^ cells) was added into the upper chamber of a 24-well transwell containing Matrigel (BD, USA) with 8 μm pores. Meanwhile, 600 μL of 1640 medium containing 10% FBS was added into the lower chamber. The plates were then incubated for 24 hours. Three replicate wells were conducted per group. After incubation, the medium was removed from the upper chamber. The cells, which had migrated to the lower surface of the membrane, were stained with 0.1% crystal violet for 5 minutes. Photomicrographs were captured and the number of cells that invaded through the membrane was counted.

### Flow cytometry analysis for apoptosis determination

After transfection with shRNA for 48 hours, U937 cells were maintained in serum-free medium to starve for 24 hours under normal conditions. Cells were made into a suspension with 1×binding buffer at 1–5×10^6^ cells/mL. Annexin V-FITC/PI was added into the cell suspensions, which was then incubated in the dark for 5 minutes at room temperature. After adding 10 uL dye liquor, the sample was detected by flow cytometry and analyzed by Flowjo software. Experiments were performed during three independent times.

### Statistical analyses

The results were displayed as mean ± SD and were analyzed using SPSS v18.0 (SPSS Inc., Chicago, IL, USA). Statistical analysis of each cell group was performed using the *T*-test. Differences with *P* < 0.05 were considered significant.
